# The Relationship between Prevention and Panic from COVID-19, Ethical Principles, Life Expectancy, Anxiety, Depression and Stress

**DOI:** 10.3390/ijerph19105841

**Published:** 2022-05-11

**Authors:** Mahdi Salehi, Grzegorz Zimon, Ali Reza Ghaderi, Zinab Ahmed Hasan

**Affiliations:** 1 Department of Economics and Administrative Sciences, Ferdowsi University of Mashhad, Mashhad 9177948974, Iran; 2 Department of Finance, Banking, and Accountancy, Faculty of Management, Rzeszow University of Technology, 35-959 Rzeszow, Poland; 3 Department of Clinical Psychology, Ferdowsi University of Mashhad, Mashhad 9177948974, Iran; a-ghaderi@um.ac.ir; 4 Department of Economics and Administrative Sciences, Imamreza International University of Mashhad, Mashhad 9137913316, Iran; zainabalhindawi@gmail.com

**Keywords:** COVID-19, ethical principles, life expectancy, anxiety, depression, stress

## Abstract

The present study aims to assess the relationship between prevention and panic from COVID-19, ethical principles, life expectancy, anxiety, depression, and stress in auditors and financial managers of small- and medium-sized Iraqi firms. In other words, this paper seeks to answer the question of whether different types of prevention and panic from COVID-19 can enhance the ethical principles, life expectancy, anxiety, depression, and stress, or not. The study method is practical in its objective and descriptive survey procedure. The study’s statistical population includes 185 employed auditors in audit firms, and 215 financial managers of small- and medium-sized Iraqi firms were selected as a sample of the study using the Cochran Sampling Method. In this paper, PLS tests are used to assess the effect of independent variables on the dependent variable. The results indicate no significant relationship between prevention from COVID-19 and ethical principles and life expectancy. However, the association between prevention from COVID-19 and anxiety, depression, and stress, and between panic from COVID-19 and ethical principles, life expectancy, anxiety, depression, and stress is positive and significant. The higher the panic from COVID-19, the more ethical principles, life expectancy, anxiety, depression, and stress. Since no study has been carried out so far on the effect of prevention and panic from COVID-19, ethical principles, life expectancy, depression, and stress in Iraqi firms, the present study results can provide valuable information and contribute to the development of science and knowledge.

## 1. Introduction

COVID-19 has accelerated dramatically since January 2020, infecting more than 373 million people worldwide and causing the deaths of more than 5 million people worldwide [1]. This pandemic led to a dramatic shift in the way people lived, worked, and played. Around the world, many companies, especially those belonging to the SME sector, have suffered enormous financial losses, have lost liquidity, or have even gone bankrupt [2,3,4].

As the world economy collapsed, families could not see their loved ones because air travel was also disrupted by the COVID [5]. As a result, governments and public health officials worldwide have provided guidelines to help smooth the curve [6]. Forcefully or voluntarily, governments issued three standard guidelines for staying home [7,8,9], using a mask [10,11,12] and observing social distancing when attending public gatherings [13].

Unfortunately, there have been instances where individuals have abandoned such regulations to protect their health and society [14]. It is not known yet what will come next in this challenge and how long we will have to endure this crisis, but all people are preparing for what will likely be a marathon rather than a sprint [14]. As a result, it is essential to understand why people choose to ignore regulations designed to protect public health. Because this will increase the prevalence of this disease and the resulting deaths, which will increase the fear and panic among the people, it is necessary to develop a framework for understanding the acceptance or resistance to these measures, including examining the ethical foundations of individuals. Ethical foundations [15,16,17] examine and judge how people behave appropriately and rightly in the face of misbehaviour. The premise of ethical foundations is based on the belief that people judge ethics intuitively and without awareness [15,18,19]. In addition, a commercial and clinical activity involving the marketing and administration of products for COVID-19 raises numerous ethical concerns and could have various harmful effects [20].

Conscious reasoning about ethics means that intuition is thought to be followed in justifying or explaining one’s intuitive ethical judgments. Thus, like many other psychological processes, judgments about ethics are made with a “dual process system” [21], according to which intuition is preferred to explicit thinking.

The COVID-19 pandemic has raised important ethical issues at various levels, such as respect for quarantine rules and sacrifice for the common good; saving the economy or human life (choosing patients for the first time for treatment); continuing working activities, and endangering the health of loved ones; deciding to return to one’s hometown at risk of an outbreak, especially during a pandemic [22,23]. Because ethical problems and ethical distress are often inevitable [22], an ethical dilemma is a problematic situation involving a conflict between two unique reciprocal options, showing both negative and undesirable consequences [24,25,26]. The COVID-19-pandemic has brought many ethical issues into a new, urgent light. A significant body of literature is available on ethical concerns in pandemics and a variety of severe ethical problems throughout the pandemic [23]. This is a situation in which a person is confronted with two ethical principles, as opposed to each other, which involve maximizing the public interest based on a benefit analysis or deciding on unconditional respect for the law, regardless of the consequences. Ethical distress occurs when individuals know which option is ethically appropriate but cannot choose due to internal or external constraints [23,27,28]. In emergencies such as pandemics, some decisions are made under stress, and several studies have shown that stress can affect ethical decision making [29,30,31,32,33]. The COVID-19 pandemic does not fit into prevailing post-traumatic stress disorder (PTSD) models or diagnostic criteria, yet emerging research shows traumatic stress symptoms resulting from this ongoing global stressor. Current pathogenic event models focus on past and largely direct trauma exposure to certain kinds of life-threatening events. Yet, traumatic stress reactions to future, indirect trauma exposure, and non-Criterion A events exist, suggesting COVID-19 is also a traumatic stressor which could lead to PTSD symptomology [34]. Francis and McNab [34] noted that the COVID-19 pandemic has led to fundamental changes in social, health, and political measures based on what is right or wrong and affects the basic ethical principles of decision-making processes. In addition, ethically inspired public messages have increased significantly during the pandemic [35]. These public messages from government agencies, celebrities, and health officials encourage citizens to adopt certain behaviours as ethical imperatives based on utilitarian, virtuous, or deontological ethical theories [36,37,38,39]. Ethical judgment and socio-cognitive abilities, especially the Theory of Mind and empathy, are closely related [37,40,41,42,43,44,45,46]. According to the dual-process model [47,48,49], ethical decision-making involves cognitive and emotional conflict processes. Cognitive processes, which are relatively slow and based on consultative reasoning, while the emotional processes are rapid and automated, operate independently of cognitive resources and advocate for deontological solutions [48,49,50]. This evidence supports the hypothesis that ethical decision-making involves socio-cognitive processes [51,52,53]. Although the influence of emotion on individuals’ ethical decisions has been identified, little is known about how emotions influence individuals’ ethical decision processes. Thus, it is unclear whether different emotions promote and/or discourage ethical decision making in the workplace [54].

Some recent studies have examined the effect of COVID-19 on empathy [55,56,57] and the psychological consequences [58,59,60,61]. Within a few months, COVID-19 disrupted the lives of virtually everyone and caused tremendous anxiety, trauma, and grief [62]. In our view, there is a research gap in that no study has focused on specific individuals who have undergone various pandemic changes. To assess the fear and panic caused by COVID-19 disease, the fear and panic questionnaire is used, which has been applied in different countries such as Iran [63], Bangladesh [64], Turkey [65], Russia and Belarus [66], Israel [67,68] Peru [69], and Paraguay [70]. Further, the Schneider standard questionnaire will measure the life expectancy standard, and the DASS21 measure anxiety, depression, and stress. Using the Anxiety and Depression Scale [71], most of these studies showed that there is a significant relationship between fear and panic regarding COVID-19 with anxiety and, to some extent, depression [72]. Recent studies have shown a significant relationship between fear of COVID-19 and anxiety, stress, and depression [61]. Moreover, some studies have shown that there is a significant negative relationship between life expectancy and fear of COVID-19, so according to Mamun and Griffiths [72], there is a significant relationship between suicide and fear of COVID-19, and in some countries, people commit suicide for fear of this disease.

Furthermore, the high daily rate of new deaths and the information citizens receive through the media can affect mood disorders [66,73,74,75]. Huang and Zhao [74] also showed a significant relationship between fear of COVID-19 and stress, anxiety, and depression in China.

The pleasure that people experience from their work results from the many efforts and conflicts; work can also have negative effects. It has been found that social stressors at work and aggression, anger, and jealousy in the workplace have serious negative consequences for employees, organisations, and society. For example, intrinsic traits can have a negative impact on the performance of individuals (such as students, employees of a business unit, etc.). Research has also shown that stressors and aggression in the workplace can negatively affect performance and organisational innovation. Hence, factors such as the spread of dangerous diseases such as COVID-19 can have a negative impact on the functioning of all members of society. Therefore, the innovation of the present research is that researching this subject can provide useful information to all. In addition, since pandemic responses have shown a significant body of literature on ethical concerns in pandemics [76], these insights have not been considered broadly. Therefore, the present study will examine the relationship between the prevention and panic of COVID-19, ethical principles, life expectancy, anxiety, depression, and stress in auditors and financial managers of small- and medium-sized companies.

## 2. Theoretical Foundations and Hypotheses Development

The prevalence of COVID-19 in 2019 can be stressful for people. Fear and worry about a disease can be overwhelming and cause intense emotions in adults and children. Coping with stress makes you, the people you care about, and your community stronger.

Depression is one of the main causes of disability in modern societies [74,75]. The experience of natural disasters increases long-term levels of depression in individuals [77,78,79]. It may also increase the rate of suicide in the future [80]. Increasing the experience of traumatic events in life and the difficulty of coping with them are among the factors that increase depression, anxiety, and stress in individuals [81].

The world is currently in a critical state caused by COVID-19, which has significantly increased depression, stress, and anxiety in different countries. The inability to fight COVID-19 in some countries affected by the virus, such as those in Latin America, is a great concern. During the coronavirus crisis, studying the causes of depression, stress, and shock and ways to prevent and fear it in accounting students in undergraduate and graduate courses and auditors working in Iraq and Iran can be of strategic importance to reduce and prevent this disease in the future.

The pandemic has forced many governments to enact strict laws to prevent its spread [79,80]. Governments in affected countries have long been isolated regarding the number of infections, illnesses, and deaths, such as China, Italy, Spain, and Ecuador, where citizens stay at home. This has seriously affected people’s living conditions. This is especially dangerous and worrying in countries with fewer resources, such as in countries in the Latin American region. Certain aspects of the disease, such as uncertainty about how it spreads, how it develops, immunity from infected patients, or the lack of a vaccine against the disease, have increased fear [81,82,83,84,85,86]. In what follows, we will explain how the prevention and panic of COVID-19 affect ethical foundations, life expectancy, anxiety, depression, and stress.

### Explaining the Relationship between COVID-19 Prevention and Panic, Ethical Principles, Life Expectancy, Anxiety, Depression, and Stress

The COVID-19 pandemic has created a rapidly evolving and threatened situation. Recent reports have shown that the coronavirus pandemic is significantly associated with the risk of mental disorders (e.g., schizophrenia, anxiety, depression, anxiety, acute stress disorder, and suicide) among health care professionals and the general public [87,88,89,90,91,92]. Similar pandemics (such as SARS) have had serious negative consequences on mental health and have mainly caused anxiety and depressive disorders [89,90]. For example, in the early stages of the SARS pandemic, a wide range of people developed mental illnesses, including persistent depression, anxiety, panic attacks, motor excitement, symptoms of psychosis, delirium, and even suicide [88,92]. Therefore, COVID-19 can significantly affect people’s daily emotional experiences. In most cases, no specific cause for anxiety can be found, and it is caused by a set of biological, psychological, and social factors. Studies show that heredity also plays a role in the development of anxiety [91]. Along with psychological and biological factors that allow anxiety in humans, the role of social factors should not be ignored. Being in a certain social situation, especially if that situation plays a decisive role in a person’s current or future life, naturally increases anxiety.

Responding to COVID-19 threats and the public health measures taken to assist it has slowed the transmission of the COVID-19 virus and may lead to a wide range of negative emotions that take a specific form [93,94,95,96]. Fear appears to be an emotional response to imminent threats such as COVID-19 [97,98]. Bavel et al. [99] noted that fear might be a significant emotional issue caused by a pandemic. Negative emotions from the threat of an pandemic can be contagious and may relate people’s feelings about others [92]. Excessive fear of COVID-19 can worsen stress, anxiety, and depression [82,100]. Negative psychological effects of public health measures have also included confusion and anger [39]. Therefore, the discrete emotional approach may be instructive in describing the psychological impact of the COVID-19 pandemic in comparison with general emotional well-being measures.

During an outbreak, community anxiety can increase following the first death, increasing media coverage and increasing the number of new deaths [60,95]. Continued exposure to COVID-19 in print, on video, and on social media can also increase anxiety and fear among people. Public health measures and their consequences (e.g., job loss, financial insecurity, and disruption of daily activities) are likely to have a negative impact on mental health [60,92]. Most studies on psychological consequences and interventions related to COVID-19 have focused on the risk factors for mental health problems [80,81,82,83,84,85,86]. Challenging stressors are desirable opportunities that lead to growth and development. Such factors, despite boring, cause positive emotions such as pride, passion, and excitement [76]. The negative impact and emotional responsibility have also been the main reasons for the risk of clinical and emotional problems related to the pandemic in Italy [94]. Fear of the pandemic, impatience, frustration, anger, and symptoms of post-traumatic stress and avoidant behaviours were found to be stressors in quarantine [39] and can affect patients with mental health problems [81] because emotional responses are part of the COVID-19 stress response (such as fear) [89], the ability to perceive and regulate one’s emotional experiences may be considered as a protective body. On the other hand, ethical analysis can occur early, should a crisis emerge. Ethical considerations need to be proactively considered in the COVID-19-pandemic [101,102,103,104,105]. Therefore, in the present study, we expect that the fear of COVID-19 will increase depression, stress, anxiety, and distress and decrease the life expectancy of accounting students, auditors, and financial managers of small and medium enterprises in Iraq. Therefore, according to what has been said, the research hypotheses are as follows:

**Hypothesis** **1** **(H1).** *There is a significant relationship between the prevention of COVID-19 and ethical principles.*

**Hypothesis** **2** **(H2).** *There is a significant relationship between the prevention of COVID-19 and life expectancy.*

**Hypothesis** **3** **(H3).** *There is a significant relationship between the prevention of COVID-19 and anxiety.*

**Hypothesis** **4** **(H4).** *There is a significant relationship between the prevention of COVID-19 and depression.*

**Hypothesis** **5** **(H5).** *There is a significant relationship between the prevention of* *COVID-19* *and stress.*

**Hypothesis** **6** **(H6).** *There is a significant relationship between the panic of* *COVID-19* *and ethical principles.*

**Hypothesis** **7** **(H7).** *There is a significant relationship between the panic of* *COVID-19* *and life expectancy.*

**Hypothesis** **8** **(H8).** *There is a significant relationship between the panic of* *COVID-19* *and anxiety.*

**Hypothesis** **9** **(H9).** *There is a significant relationship between the panic of* *COVID-19* *and depression.*

**Hypothesis** **10** **(H10).** *There is a significant relationship between the panic of* *COVID-19* *and stress.*

## 3. Methodology

The present study is practical in terms of the objective and type of study, and it is a survey method in terms of analysis of the collected data. This method is referred to as the field method; the scholar will collect the data and information by being present at the statistical population level and utilising different tools, including a questionnaire. The survey method is used for assessing the distribution of statistical population features to control the status quo and explore the relationship between events. The collected data will be analysed via R Statistic Software. The reliability of the study results will be examined using the gathered information, and the obtained results can be generalised to the entire statistical population.

The information used in this paper is divided into two sources. First, the literature about COVID-19, ethical principles, life expectancy, anxiety, depression, and stress was reviewed. Second, information was collected from the questionnaire. The research questionnaire is based on the standard questionnaires of different intelligence and occupational performance questionnaires, many of which are omitted regarding a large number of questions and the inappropriateness of some questions with the status quo in Iraq. This study was carried out in 2020 on the Iraqi Stock Exchange in the audit firm section. The respondents are faced with two parts; by answering the first part, we can figure out whether or not the factor is currently present in Iraqi audit firms considering the professional experience of the respondent. By answering the second part, the amount of significance (extremely high, high, medium, low, extremely low) will be expressed from the respondents’ point of view. The opinion of opinion leaders assesses the validity of the questionnaire, and the reliability is examined using Cronbach’s Alpha.

### 3.1. Population and Sample

The study’s statistical population includes all auditors and financial managers of small- and medium-sized Iraqi firms during 2020. In this paper, the auditors and financial managers of small- and medium-sized Iraq firms are studied as the study sample. In other words, the sampling method in this paper is simple randomising, such that the sample members are selected randomly, and the questionnaires are distributed among them. The sampling method of the present study is based on a Cochran and Morgan Table and finally, 185 employed auditors in audit firms and 215 SME managers were selected as the sample of the study.

The study’s statistical population included 402 auditors working in auditing firms and financial managers of small and medium enterprises in Iraq. A total of 198 were selected by the Cochran sampling method as the sample size. Due to the COVID-19 situation in Iraq, and because employees work remotely during the time of COVID-19 and the research population is large, direct access to members has been difficult. For this purpose, 260 questionnaires were distributed in hard copy, and also online questionnaires were considered. During the 45 days, 123 online questionnaires were completed, and 75 hard-copy questionnaires were received, for a total of 198 people who answered the questionnaire questions, so the participation rate is 76%.

In this study, PLS tests were used to investigate the effect of independent variables on the dependent variable.

### 3.2. Research Model and Variables

**Figure 1:**
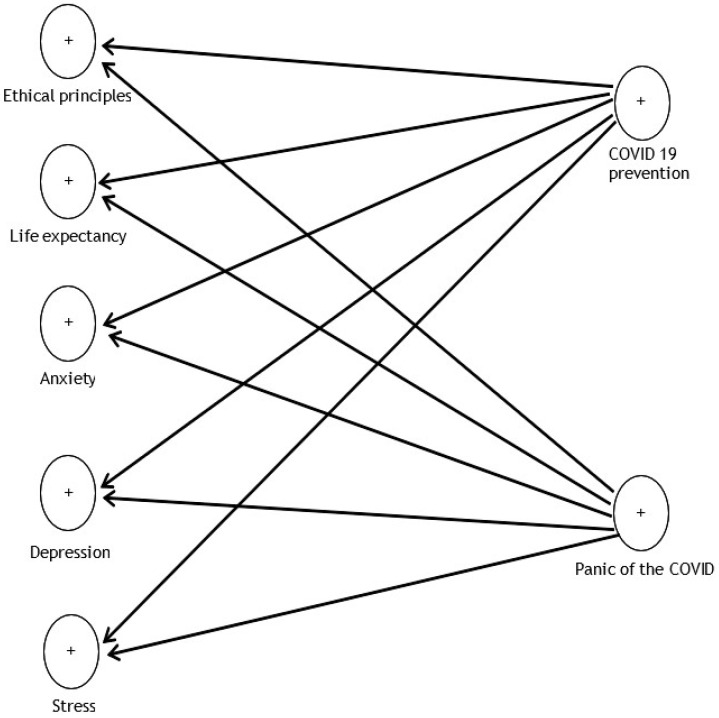
Research model.

The measurement model is that part of the model that includes a variable and some related items. This paper has seven prevention models: COVID-19, panic from COVID-19, ethical principles, life expectancy, anxiety, depression, and stress. The model is presented in Figure 1.

The variables used in the present study are the questions posed in the questionnaires. These questionnaires include emotional, spiritual, and organisational intelligence, social capital, and organisational performance that are classified in five points (Likert scale) from (1) strongly disagree to (5) strongly agree and are defined as follows:

To assess the fear and panic caused by COVID-19 disease, the fear and panic questionnaires in different countries such as Bangladesh, Italy, Turkey, Russia and Belarus, Israel, Peru, and Paraguay have been used [64,65]. The standard questionnaire was used to measure life expectancy. The standard Beck Anxiety Inventory (2003) questionnaire measured anxiety. Beck Depression Inventory (2003) was used to assess depression. The Beck standard stress questionnaire (2003) was used to measure stress.

**COVID:** COVID-19 virus spreads easily in some geographical regions.

**Depression:** in this paper, there are some scores the respondents obtain from the Beck Depression Inventory, second edition (BDI-II). The questionnaire includes 21 questions covering all depression elements based on the cognitive theory.

**Life expectancy:** by life expectancy, we mean the score the respondent obtains from the Miller life expectancy questionnaire that includes 48 questions, in a way that 48 is distressed and 240 is for maximum expectancy.

**Ethical principles:** in this paper, professional ethics is the respondents’ score from 25 questions of the professional ethics questionnaire. The questionnaire was designed in 2002 by Kadozir to assess professional ethics. It contains 25 questions and eight components of regulating values, honesty, sympathy with others, responsibility, justice and fairness, loyalty, superiority, and respecting others, that examine the professional ethics based on the five-point Likert scale, with questions like, to what extent do you respect and implement your beliefs in doing actions?

**Anxiety:** in this paper, anxiety is an individual’s score from the Spielberger State-Trait Anxiety Inventory (STAI).

In this paper, stress is the score an individual achieves from the Holmes–Rahe Stress Inventory.

The variables used in the present study are the questions that have been asked in the questionnaires of the present study. These questionnaires include the COVID Prevention and Panic Questionnaire based on ethics, life expectancy, anxiety, depression and stress, classified in five points (Likert scale) from strongly agree to strongly disagree.

To assess the quality of the measurement model, a study should examine the validity and reliability of different concepts and variables. Cronbach’s alpha reliability is used in survey studies to assess the internal errors of indicators of a variable. The method is one of the most prevalent traditional techniques for examining internal homogeneity among indicators since it is assumed that all indicators of external loads enjoy the same load. The index for total reliability of the scale is a statistic named alpha, the interval of which is between zero and one. The cutting point is 0.7. In other words, the higher the alpha coefficient, the higher the reliability scale would be. In this paper, Cronbach’s alpha values for all structures enjoy an appropriate value.

The combined reliability value for prevention from COVID-19 is 0.805, panic from COVID-19 0.892, ethical principles 0.782, life expectancy 0.751, anxiety 0.903, depression 0.744, and stress 0.825. Table 1 shows the combined reliability of the research model variables.

Convergent validity is the second criterion for fitting measurement models in the PLS method. The average variance extracted (AVE) criterion is indicative of the average shared variance between each structure and its indicators, and the higher the correlation, the higher the fitting. Fornell and Larcker introduce the AVE criterion to measure convergent validity and state that the critical value is 0.5, which means an AVE value higher than 0.5 shows an acceptable convergent validity [80]. The AVE value higher than 0.4 is sufficient, but for upcoming more accurate calculations, it is better to set the value at 0.5.

**Figure 2:**
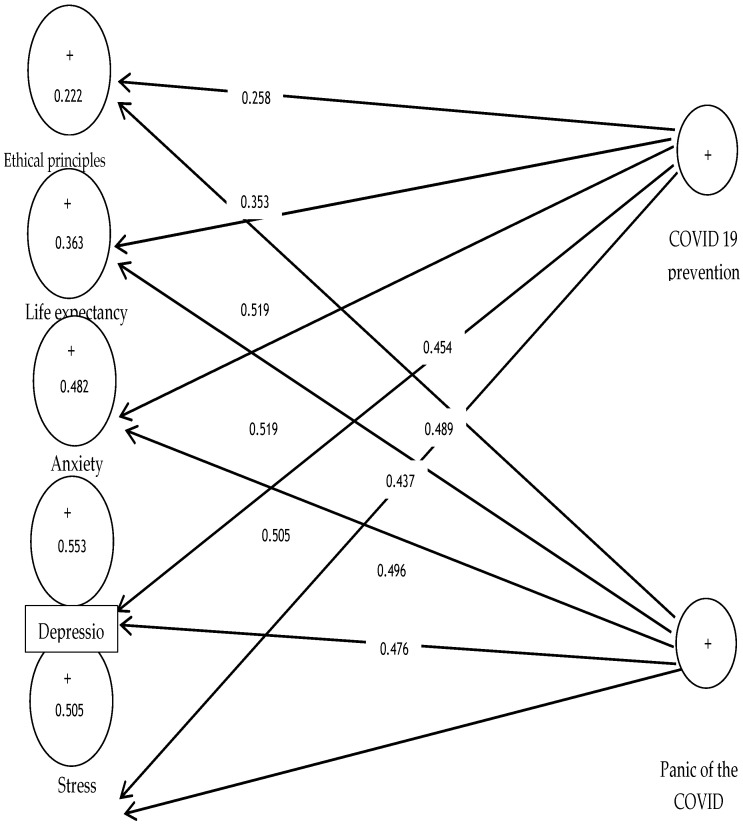
Factor load values.

As shown in Figure 2, factor load values are favourable in the path coefficient analysis. All factor load values are more than 0.4 except for ethical principle structures and life expectancy with factor loads of 0.258 and 0.353, with no acceptable values.

Divergent validity is a value that distinguishes a variable from another one in terms of experimental criteria. In other words, this type of credit is expected from the correlation of a variable to be more than that of other variables. According to the variance-based approach, there are two general criteria for evaluating divergent validity:

The Fornell and Larcker method is the second scale for assessing divergent credit and compares the second root of AVE values with the correlation of other hidden variables. The rationale behind the method is based on the idea that a variable has more variance in common with its modifiers than other variables. The AVE scale calculates the average variance extracted from a variable. Table 2 shows the divergent validity matrix of the research model by the Fornell–Larker method.

The main diameter of the matrix is the root of AVE values for stress (0.721), anxiety (0.708), depression (0.632), life expectancy (0.615), ethical principles (0.608), and panic of COVID-19 (0.604), and prevention from COVID-19 (0.588). As can be seen in Table 3, the value of root AVE of the stress structure (0.721) is higher than the correlation value of the six structures of anxiety, depression, life expectancy, ethical principles, panic from COVID-19, and prevention from COVID-19 (0.449, 0.671, 0.611, 0.276, 0.627, 0.469). That is the same for the structures of anxiety, depression, life expectancy, ethical principles, panic from COVID-19, and prevention from COVID-19. Hence, we can declare that the model’s structures (hidden variables) interact more with their indicators than other structures in the present study. In other words, the divergent validity of the model is appropriate.

According to the data analysis algorithm in the PLS method, we turn it into structural model fitting after analysing measurement model fittings. In contrast to measurement models, the structural model section is not about questions (explicit variables) and only examines the hidden variables and their relations.

For the analysis of t significance figures, the minimum acceptable value for the criterion is 1.96. When the t value for external weights of each item is more than 1.96, we can say that the external weights for the item of the measurement model in the structure are confirmed at a 95% confidence level.

**Figure 3:**
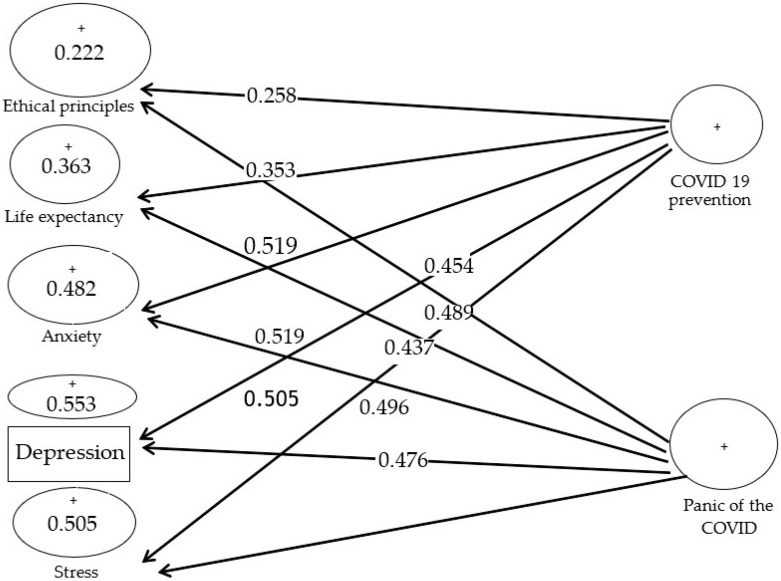
The t significance coefficients.

Figure 3 is the output of the conceptual framework along with t significance coefficients, and if the value is 1.96 for a path, the path is confirmed at a 95% confidence level. All indicators of seven conceptual models, except two ethical principles and life expectancy structures, are higher than 1.96 to explain the related structures appropriately.

The most important values used for estimating the structural model are the determination coefficient that indicates model prediction. The coefficient is achieved from the square of the relationship of endogenous variables with predictive variables. In other words, the path of the coefficient of determination within a model shows the value of the explained variance of the endogenous hidden variable obtained from the effect of an exogenous hidden variable on an endogenous one, so it calculates for endogenous hidden variables. The R^2^ coefficient of determination is a criterion for linking the measurement section and the structural section of structural equation modelling and is indicative of the effect an exogenous variable has on an endogenous one, for which three values of 0.67, 0.33, and 0.19 are considered as the criterion values for weak, medium, and strong values. The R^2^ value calculates only for endogenous (dependent) structures, zero for exogenous structures. Table 3 shows the values of R^2^.

According to Table 3, the coefficient of determination for life expectancy, anxiety, depression, and stress is medium. The value is weak for the variable of ethical principles, showing that the effect of prevention from COVID-19 and panic from COVID-19 on the endogenous variable is medium.

The severity of the prediction power of the model about endogenous structures has three values of 0.35, 0.15, and 0.02.

The outputs of the software for the redundancy index of data analysis show that the obtained values for the variables of stress, anxiety, and depression, with values of 0.206, 0.261, and 0.197, reveal a medium prediction power of the redundancy index and the variables of life expectancy and ethical principles have a weak prediction power. As for the panic structures from COVID-19 and prevention from COVID-19, the Q2 criterion value is zero since the structures are exogenous. We can claim that the study’s conceptual framework enjoys an appropriate prediction power.

In the goodness of fit of the research model, the mean of commonality values is achieved from seven variables: stress, anxiety, depression, life expectancy, ethical principles, panic from COVID-19, and prevention from COVID-19.

$$\bar{Communality}=(0.520+0.574+0.500+0.502+0.528+0.541+0.514)/7=0.525$$

The R^2^ value for the structures of ethical principles, life expectancy, anxiety, depression, and stress is equal to:

$$\bar{R}=(0.222+0.363+0.482+0.553+0.505)/5=0.425$$

So, the GOF value for the first model is:

$$\mathrm{GOF}=\sqrt{0.525*0.425}=0.308$$

The GOF criteria is a value between 0 and 1, for which Wetzels defined three values of 0.36, 0.25, and 0.01 as weak, medium, and strong values, and the higher the value, we can say that the general fit of the model is at an appropriate level. According to the obtained value of 0.308 for the criterion, the medium fit of the model is confirmed, and the study can be continued.

## 4. Results

The respondents’ information to the questionnaire, including gender, level of education, the field of study, age and level of professional experience, is presented in Table 4. The results show that 82.3% of the respondents are male, and the rest (17.7%) are female. A total of 62.6% of the respondents have a bachelor’s degree, 19.7% have a master’s degree, and 2% have a PhD. Table 4 shows that 10.6% of respondents are between 20 and 30 years old, and 8.1% are older than 50 years. In addition, 12.1% of respondents have less than five years of professional experience, 62.1% of the respondents have five to 10 years of professional experience, 8.1% of the respondents have 11 to 15 years of professional experience, and 3.5% of the respondents have more than 20 years of professional experience.

### Hypotheses Testing

**Figure 4:**
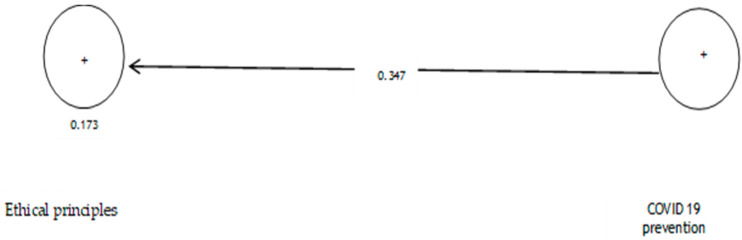
The model of the first hypothesis includes factor load coefficients.

**Figure 5:**
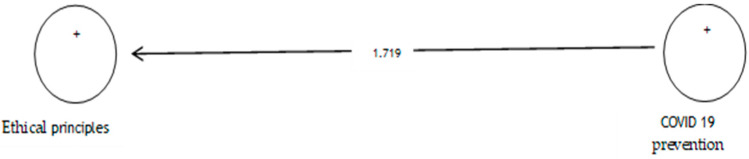
Drawn model with significant coefficients z.

The variance-based structural equation modelling is used to assess the first hypothesis. The independent and dependent variables of the study were entered into the structural equation model in the form of hidden variables and first-order factor models. The estimations related to the evaluation indicators of the general evaluation of the structural equation model and the key parameters of the model (significance of association between variables) are displayed in the following figure and table:

The analysis shows that the path coefficient between two prevention variables from COVID-19 and ethical principles is equal to 0.347 (Figure 4). On the other hand, since the t value between these two variables is equal to 1.719 (Figure 5), that is, calculated at a significant level of less than 5% (*p* = 0.086), we can claim that the hypothesis is not confirmed, so we can conclude that there is no significant relationship between prevention from COVID-19 and ethical principles. Table 5 shows the evaluation indicators of the internal model of the research, direction, and significance of direct effects.

**Figure 6:**
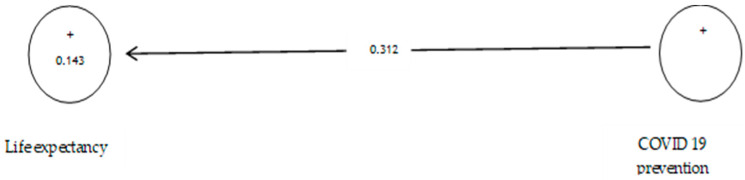
The second sub-hypothesis model includes factor load coefficients.

**Figure 7:**
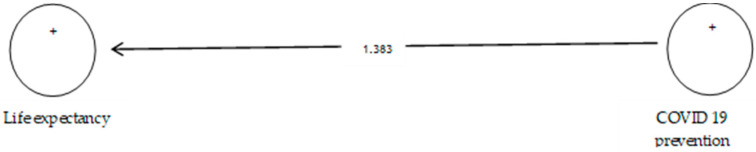
Drawn model with significant coefficients z.

The variance-based structural equation modelling is used to assess the second hypothesis. The independent and dependent variables of the study were entered into the structural equation model in the form of hidden variables and first-order factor models. The estimations related to the evaluation indicators of the general evaluation of the structural equation model and the critical parameters of the model (significance of association between variables) are displayed in the following figure and table:

According to Table 6, the analysis shows that the path coefficient between two prevention variables from COVID-19 and life expectancy equals 0.312 (Figure 6). On the other hand, since the t value between these two variables is equal to 1.383 (Figure 7), that is, calculated at a significant level of less than 5% (*p* = 0.066), we can claim that the hypothesis is not confirmed, so we can conclude that there is no significant relationship between prevention from COVID-19 and life expectancy while according to research such as [105,106,107], life expectancy declined from 2019 to 2020.

**Figure 8:**
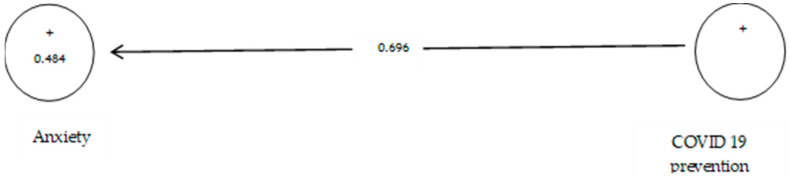
The third sub-hypothesis model includes factor load coefficients.

The variance-based structural equation modelling is used to assess the third hypothesis. The independent and dependent variables of the study were entered into the structural equation model in the form of hidden variables and first-order factor models. The estimations related to the evaluation indicators of the general evaluation of the structural equation model and the key parameters of the model (significance of association between variables) are displayed in the following figure and table:

The analysis shown in Table 7 indicates that the path coefficient between two prevention variables from COVID-19 and anxiety is equal to 0.696 (Figure 8). On the other hand, since the t value between these two variables is equal to 14.514, that is, calculated at a significant level of more than 5% (*p* = 0.0), we can claim that the hypothesis is confirmed, so we can conclude that there is a significant relationship between prevention from COVID-19 and anxiety. This conclusion may be due to anxiety about infected withCOVID-19 [108].

**Figure 9:**
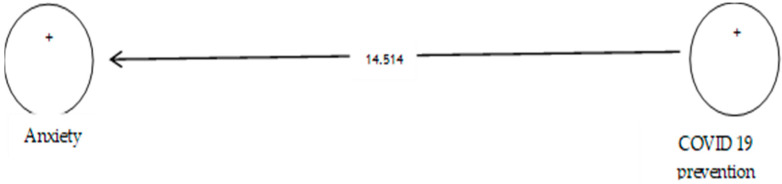
Drawn model with significant coefficients z.

The variance-based structural equation modelling is used to assess the fourth hypothesis. The independent and dependent variables of the study were entered into the structural equation model in the form of hidden variables and first-order factor models. The estimations related to the evaluation indicators of the general evaluation of the structural equation model and the key parameters of the model (significance of association between variables) are displayed in Figure 9.

**Figure 10:**
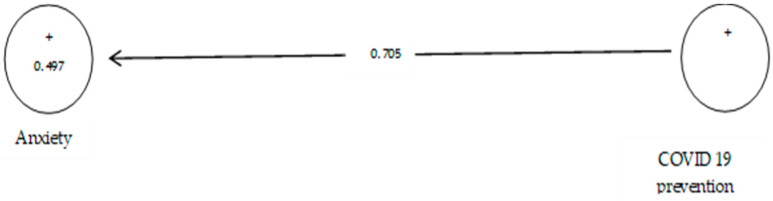
The fourth sub-hypothesis model includes factor load coefficients.

**Figure 11:**
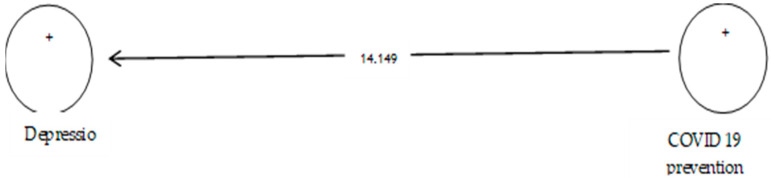
Drawn model with significant coefficients z.

According to Table 8, the analysis shows that the path coefficient between two prevention variables from COVID-19 and depression equals 0.705 (Figure 10). On the other hand, since the t value between these two variables is equal to 14.149 (Figure 11), that is, calculated at a significant level of more than 5% (*p* = 0.0), we can claim that the hypothesis is confirmed, so we can conclude that there is a significant relationship between prevention from COVID-19 and depression.

**Figure 12:**
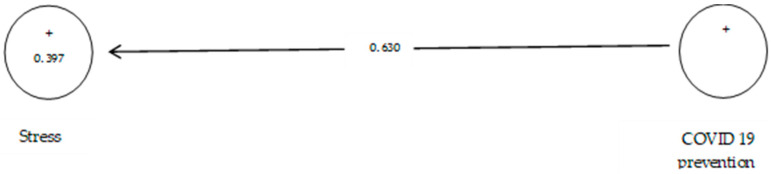
The fifth sub-hypothesis model includes factor load coefficients.

**Figure 13:**
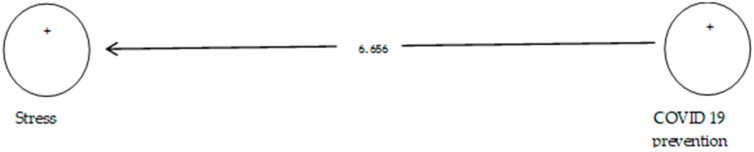
Drawn model with significant coefficients z.

The variance-based structural equation modelling is used to assess the fifth hypothesis. The independent and dependent variables of the study were entered into the structural equation model in the form of hidden variables and first-order factor models. The estimations related to the evaluation indicators of the general evaluation of the structural equation model and the key parameters of the model (significance of association between variables) are displayed in the following figure and table:

According to Table 9, the analysis shows that the path coefficient between two prevention variables from COVID-19 and stress equals 0.630 (Figure 12). On the other hand, since the t value between these two variables is equal to 6.656, (Figure 13) that is, calculated at a significant level of more than 5% (*p* = 0.0), we can claim that the hypothesis is confirmed, so we can conclude that there is a significant relationship between prevention from COVID-19 and stress.

**Figure 14:**
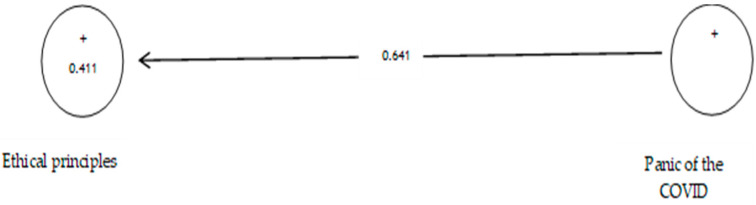
The sixth sub-hypothesis model includes factor load coefficients.

**Figure 15:**
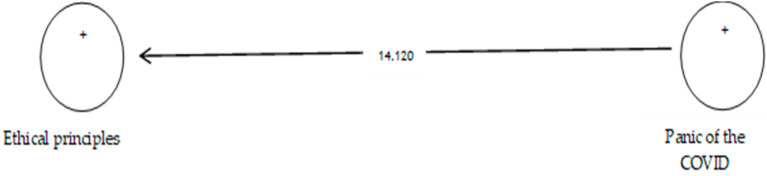
Drawn model with significant coefficients z.

The variance-based structural equation modelling is used to assess the sixth hypothesis. The independent and dependent variables of the study were entered into the structural equation model in the form of hidden variables and first-order factor models. The estimations related to the evaluation indicators of the general evaluation of the structural equation model and the key parameters of the model (significance of association between variables) are displayed in the following figure and table:

According to Table 10, the analysis shows that the path coefficient between two prevention variables from COVID-19 and ethical principles equals 0.641 (Figure 14 and Figure 15). On the other hand, since the t value between these two variables is equal to 14.120, that is, calculated at a significant level of more than 5% (*p* = 0.0), we can claim that the hypothesis is confirmed, so we can conclude that there is a significant relationship between the panic of COVID-19 and ethical principles.

**Figure 16:**
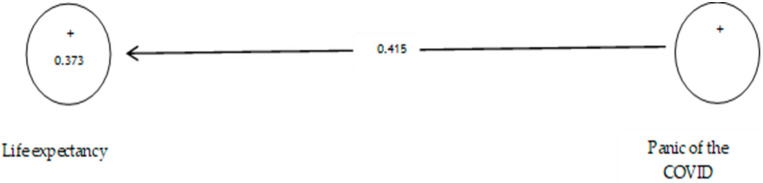
The seventh sub-hypothesis model includes factor load coefficients.

**Figure 17:**
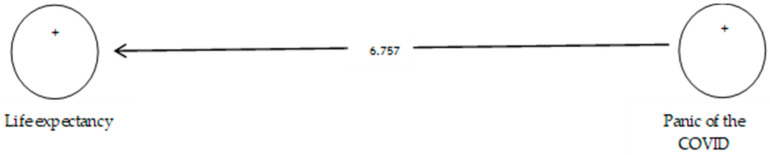
Drawn model with significant coefficients z.

The variance-based structural equation modelling is used to assess the seventh hypothesis. The independent and dependent variables of the study were entered into the structural equation model in the form of hidden variables and first-order factor models. The estimations related to the evaluation indicators of the general evaluation of the structural equation model and the key parameters of the model (significance of association between variables) are displayed in the following figure and table:

According to Table 11, the analysis shows that the path coefficient between two prevention variables from COVID-19 and life expectancy equals 0.415 (Figure 16). On the other hand, since the t value between these two variables is equal to 6.757, (Figure 17) that is, calculated at a significant level of more than 5% (*p* = 0.0), we can claim that the hypothesis is confirmed, so we can conclude that there is a significant relationship between the panic of COVID-19 and life expectancy.

**Figure 18:**
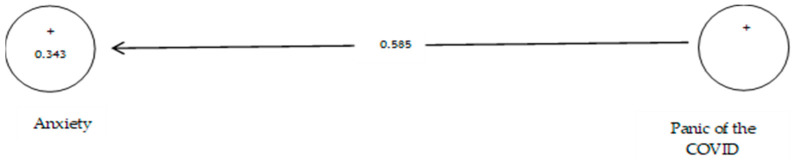
The eighth sub-hypothesis model includes factor load coefficients.

**Figure 19:**
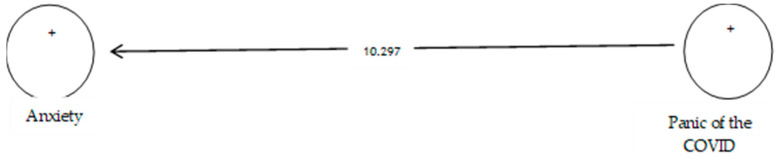
Drawn model with significant coefficients z.

The independent and dependent variables of the study were entered into the structural equation model in the form of hidden variables and first-order factor models. The estimations related to the evaluation indicators of the general evaluation of the structural equation model and the key parameters of the model (significance of association between variables) are displayed in the following figure and table:

Table 12 shows that the path coefficient between two variables of prevention from COVID-19 and anxiety equals 0.585 (Figure 18). On the other hand, since the t value between these two variables is equal to 10.297, (Figure 19) that is, calculated at a significant level of more than 5% (*p* = 0.0), we can claim that the hypothesis is confirmed, so we can conclude that there is a significant relationship between the panic of COVID-19 and anxiety. Studies showed that anxiety symptoms were much higher than before the pandemic [109].

**Figure 20:**
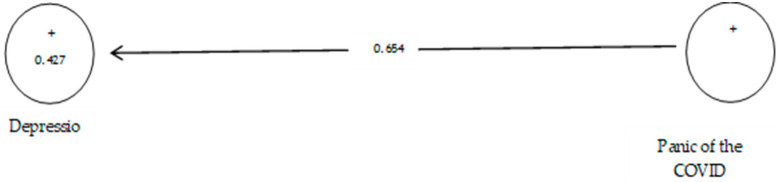
The ninth sub-hypothesis model includes factor load coefficients.

**Figure 21:**
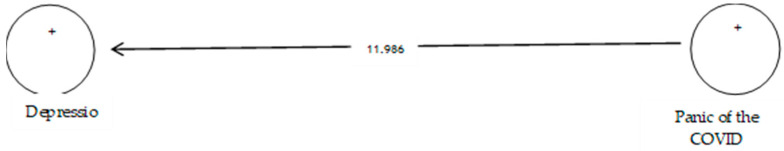
Drawn model with significant coefficients z.

The variance-based structural equation modelling is used to assess the ninth hypothesis. The independent and dependent variables of the study were entered into the structural equation model in the form of hidden variables and first-order factor models. The estimations related to the evaluation indicators of the general evaluation of the structural equation model and the key parameters of the model (significance of association between variables) are displayed in the following figure and table:

Table 13 shows that the path coefficient between two prevention variables from COVID-19 and depression equals 0.654 (Figure 20). On the other hand, since the t value between these two variables is equal to 11.986, (Figure 21) that is, calculated at a significant level of more than 5% (*p* = 0.0), we can claim that the hypothesis is confirmed, so we can conclude that there is a significant relationship between the panic of COVID-19 and depression. Studies showed symptoms of depression were at a much higher level than prior to the pandemic [108].

**Figure 22:**
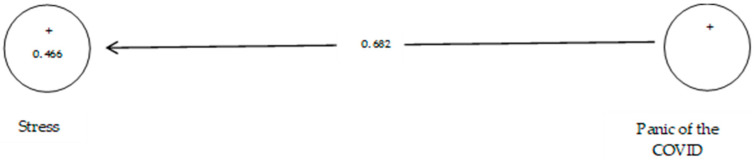
The tenth sub-hypothesis model includes factor load coefficients.

**Figure 23:**
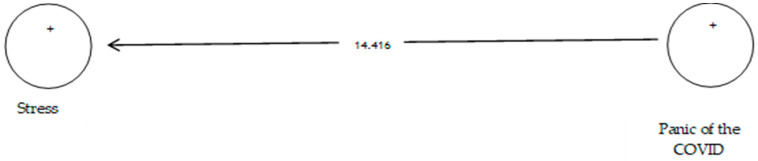
Drawn model with significant coefficients z.

The variance-based structural equation modelling is used to assess the tenth hypothesis. The independent and dependent variables of the study were entered into the structural equation model in the form of hidden variables and first-order factor models. The estimations related to the evaluation indicators of the general evaluation of the structural equation model and the key parameters of the model (significance of association between variables) are displayed in the following figure and table:

According to Table 14, the analysis shows that the path coefficient between two prevention variables from COVID-19 and stress equals 0.682 (Figure 22). On the other hand, since the t value between these two variables is equal to 14.416, (Figure 23) that is, calculated at a significant level of more than 5% (*p* = 0.0), we can claim that the hypothesis is confirmed, so we can conclude that there is a significant relationship between the panic of COVID-19 and stress.

## 5. Discussion and Conclusions

The present study assesses the relationship between the prevention and panic from COVID-19, ethical principles, life expectancy, anxiety, depression, and stress in auditors and financial managers of small- and medium-sized firms. The results of hypothesis testing show no significant relationship between prevention from COVID-19 and ethical principles and life expectancy. However, there is a positive and significant relationship between prevention from COVID-19 and anxiety, depression, and stress, and between panic from COVID-19 and ethical principles, life expectancy, anxiety, depression, and stress. The higher the panic from COVID-19, the higher the ethical principles, life expectancy, anxiety, depression, and stress. Participants in the study show a high level literature on stress, anxiety, and depression [93]. That shows a considerable proportion of psychological health issues among students is lockdown due to COVID-19, which appears with mild to severe signs of anxiety, stress, and depression in the initial stages of the pandemic [74].

The results of the study on the hypothesis of panic from COVID-19, stress, anxiety, and depression are in line with that of Huang and Zhao [74] and Sandín et al. [103], who declare that there is a positive and significant relationship between panic from COVID-19, stress, anxiety, and depression.

The obtained results propose novel knowledge since, in most of the topical literature, the levels of the studies have been about students and health workers instead of auditors and managers of small- and medium-sized firms [95]. Moreover, by analysing the topical literature, we conclude that the association discovered between the panic of COVID-19 and depression conforms with some studies, including Alyami et al. [77] and Tzur et al. [83]. Moreover, the associations between panic from COVID-19 and anxiety, and anxiety and depression, are also in line with Mertens et al., Jansson-Fröjmark and Lindblom, Fjermjestad et al.; Jacobson and Newman, Bridgland et al. confirm the results of the present study [104,105,106,107,109].

While variations across countries will exist in responding to the COVID-19 pandemic, the human rights of individuals with mental health disorders must be protected, and appropriate and safe services provided for their treatment. Moreover, the negative impact of this pandemic on government budgets should not be used as an excuse to reduce essential services for people with mental illness during or after the pandemic [110]. However, the condition in Iraq and the way people deal with the pandemic are very unfortunate. The rapid spread of the disease, a huge number of afflicted people, death toll rise, distrust of the health system, unawareness, and false information may aid the fact that auditors and managers of Iraqi SME firms are afraid of the situation. Panic is determined as a factor that affects depression, and this factor, along with anxiety a reduced life expectancy, can exacerbate the ethical principles. This study’s conclusion, related to ethical principles, life expectancy, anxiety, depression, and stress during COVID-19, can be used to reference other researchers developing ideas in response to patterns.

The COVID-19 pandemic has been a global economy and a health shock. Policy makers have had to balance strict public health measures to slow the spread of the virus against the adverse health, educational, and economic consequences of these choices. Attention has, therefore, rightly focused on the immediate mental health consequences of the pandemic, both for the general population and for people with mental illness [111]. According to this argument, the auditors and managers of SME firms, when they feel that they are more vulnerable and exposed to more risk, feel more unease since most of them have to leave their homes to continue with their living and be exposed to unfavourable conditions, so protecting their health is not an easy task. This study offers a timely and relevant contribution to the academic research about prevention and panic from COVID-19, ethical principles, life expectancy, anxiety, depression, and stress.

This study is subject to certain limitations. For example, access to research samples was difficult due to the prevalence of COVID-19 disease. It was also impossible to control other factors that affect auditors and financial managers’ anxiety, life expectancy, depression and stress. Furthermore, in this study, life expectancy is attributable to COVID-19, not total life expectancy.

## Figures and Tables

**Table 1:** The combined reliability of research model variables.

Variable	Combined Reliability Coefficient
COVID-19 prevention	0.805
Panic of COVID-19	0.892
Ethical principles	0.782
Life expectancy	0.751
Anxiety	0.903
Depression	0.744
Stress	0.825

**Table 2:** The divergent validity matrix of the research model by the Fornell–Larker method.

Structures	Stress	Anxiety	Depression	Life Expectancy	Ethical Principles	Panic of COVID-19	COVID-19 Prevention
Stress	0.721	0.000	0.000	0.000	0.000	0.000	0.000
Anxiety	0.469	0.708	0.000	0.000	0.000	0.000	0.000
Depression	0.627	0.645	0.632	0.000	0.000	0.000	0.000
Life expectancy	0.276	0.293	0.321	0.615	0.000	0.000	0.000
Ethical principles	0.611	0.444	0.535	0.273	0.608	0.000	0.000
Panic of COVID-19	0.671	0.568	0.628	0.387	0.510	0.604	0.000
COVID-19 prevention	0.449	0.570	0.571	0.338	0.548	0.519	0.588

**Table 3:** The values of R^2^.

Structure	R Square
Ethical principles	0.222
Life expectancy	0.363
Anxiety	0.482
Depression	0.553
Stress	0.505

**Table 4:** The respondents’ information.

Information	Status	No.	Percentage
Gender	Male	163	82.3
	Female	35	17.7
Education	Bachelor	124	62.6
	M.A.	39	19.7
	PhD.	4	2
Age	20 < 30	21	10.6
	31 < 40	137	69.2
	41 < 50	24	12.1
	>51	16	8.1
Job grade	Audit supervisor	12	6.1
	Senior audit supervisor	28	14.1
	Auditor	6	3
	Senior auditor	152	76.8
Professional experience	<5	24	12.1
	6 < 10	123	62.1
	11 < 15	16	8.1
	16 < 20	28	14.1
	>21	7	3.5

Resource: research findings.

**Table 5:** Evaluation indicators of the internal model of the research.

The Relationship of Variables	Path Coefficient (β)	Test Value t	Significance Level	Results
COVID-19 prevention—Ethical principles	0.347	1.719	0.086	Confirmed

**Table 6:** Evaluation indicators of the internal model of the research, direction and significance of direct effects.

The Relationship of Variables	Path Coefficient (β)	Test Value t	Significance Level	
COVID-19 prevention—Life expectancy	0.312	1.383	0.000	Confirmation

**Table 7:** Evaluation indicators of the internal model of the research, direction and significance of direct effects.

The Relationship of Variables	Path Coefficient (β)	Test Value t	Significance Level	
COVID-19 prevention—Anxiety	0.696	14.514	0.000	Confirmed

**Table 8:** Evaluation indicators of the internal model of the research, direction, and significance of direct effects.

The Relationship of Variables	Path Coefficient (β)	Test Value t	Significance Level	
COVID-19 prevention—Depression	0.705	14.149	0.000	Confirmed

**Table 9:** Evaluation indicators of the internal model of the research, direction and significance of direct effects.

The Relationship of Variables	Path Coefficient (β)	Test Value t	Significance Level	
COVID-19 prevention—Stress	0.630	6.656	0.000	Confirmed

**Table 10:** Evaluation indicators of the internal model of the research, direction and significance of direct effects.

The Relationship of Variables	Path Coefficient (β)	Test Value t	Significance Level	
Panic of COVID-19—Ethical principles	0.641	14.120	0.000	Confirmed

**Table 11:** Evaluation indicators of the internal model of the research, direction and significance of direct effects.

The Relationship of Variables	Path Coefficient (β)	Test Value t	Significance Level	
Panic of COVID-19—Life expectancy	0.415	6.757	0.000	Confirmed

**Table 12:** Evaluation indicators of the internal model of the research, direction and significance of direct effects.

The Relationship of Variables	Path Coefficient (β)	Test Value t	Significance Level	
Panic of COVID-19—Anxiety	0.585	10.297	0.000	Confirmed

**Table 13:** Evaluation indicators of the internal model of the research, direction and significance of direct effects.

The Relationship of Variables	Path Coefficient (β)	Test Value t	Significance Level	
Panic of COVID-19—Depression	0.654	11.986	0.000	Confirmed

**Table 14:** Evaluation indicators of the internal model of the research, direction and significance of direct effects.

The Relationship of Variables	Path Coefficient (β)	Test Value t	Significance Level	
Panic of COVID-19—Stress	0.682	14.416	0.000	Confirmed

## References

[1] Worldometer 2022. COVID-19 Coronavirus Pandemic. https://www.worldometers.info/coronavirus/.

[2] Clampit J., Hasija D., Dugan M., Gamble J. (2021). The Effect of Risk, R&D Intensity, Liquidity, and Inventory on Firm Performance during COVID-19: Evidence from US Manufacturing Industry. J. Risk Financ. Manag..

[3] Zimon G., Tarighi H. (2021). Effects of the COVID-19 Global Crisis on the Working Capital Management Policy: Evidence from Poland. J. Risk Financ. Manag..

[4] Sadowski A., Galar Z., Walasek R., Zimon G., Engelseth P. (2021). Big data insight on global mobility during the Covid-19 pandemic lockdown. J. Big Data.

[5] Garfin D.R., Silver R.C., Holman E.A. (2020). he novel coronavirus (COVID-2019) outbreak: Amplification of public health consequences by media exposure. Health Psychol..

[6] Anderson R.M., Heesterbeek H., Klinkenberg D., Hollingsworth T.D. (2020). How will country-based mitigation measures influence the course of the COVID-19 epidemic?. Lancet.

[7] Jia J.S., Lu X., Yuan Y., Xu G., Jia J., Christakis N.A. (2020). Population flow drives Spatio-temporal distribution of COVID-19 in China. Nature.

[8] Chen B., Sun J., Feng Y. (2020). How have COVID-19 isolation policies affected young people’s mental health?—Evidence from Chinese college students. Front. Psychol..

[9] Wu C., Chen X., Cai Y., Zhou X., Xu S., Huang H., Song J. (2020). Risk factors associated with acute respiratory distress syndrome and death in patients with coronavirus disease 2019 pneumonia in Wuhan, China. JAMA Intern. Med..

[10] Feng S., Shen C., Xia N., Song W., Fan M., Cowling B.J. (2020). Rational use of face masks in the COVID-19 pandemic. Lancet Respir. Med..

[11] Horwell C.J., McDonald F. The Conversation; 2020, May 5. Why You Need to Wear a Face Mask in France, but Not in the UK. https://theconversation.com/coronavirus-why-you-need-to-wear-a-face-mask-in-france-but-not-in-the-uk-137856.

[12] Barcenilla-Guitard M., Espart A. (2021). Influence of Gender, Age and Field of Study on Hand Hygiene in Young Adults: A Cross-Sectional Study in the COVID-19 Pandemic Context. Int. J. Environ. Res. Public Health.

[13] Lewnard J.A., Lo N.C. (2020). Scientific and ethical basis for social-distancing interventions against COVID-19. Lancet Infect. Dis..

[14] Precel N., Colangelo A. (2020). “That Is a Concern”: People Flout distaNcing Guidelines over Easter. https://www.theage.com.au/national/victoria/that-is-a-concern-people-flout-distancing-guidelines-over-easter-20200411-p54j2o.html.

[15] Graham J., Haidt J., Nosek B.A. (2009). Liberals and conservatives rely on different sets of moral foundations. J. Personal. Soc. Psychol..

[16] Strimling P., Vartanova I., Jansson F., Eriksson K. (2019). The connection between moral positions and moral arguments drives opinion change. Nat. Hum. Behav..

[17] Waytz A., Iyer R., Young L., Haidt J., Graham J. (2019). Ideological differences in the expanse of the moral circle. Nat. Commun..

[18] Haidt J. (2001). The emotional dog and its rational tail: A social intuitionist approach to moral judgment. Psychol. Rev..

[19] Hauser M., Cushman F., Young L., Kang-Xing Jin R., Mikhail J. (2008). A dissociation between moral judgments and justifications. Mind Lang..

[20] Major B., Schmader T., Major B., Dovidio J.F., Link B.G. (2018). Stigma, Social Identity Threat, and Health. The Oxford Handbook of Stigma, Discrimination and Health.

[21] Kahneman D.A. (2003). Perspective on judgment and choice: Mapping bounded rationality. Am. Psychol..

[22] Dunham A.M., Rieder T.N., Humbyrd C.J. (2020). A bioethical perspective for navigating moral dilemmas amidst the COVID-19 Pandemic. J. Am. Acad. Orthop. Surg..

[23] Turner L., Munsie M., Levine A.D., Ikonomou L. (2021). Ethical issues and public communication in the development of cell-based treatments for COVID-19: Lessons from the pandemic. Stem Cell Rep..

[24] Sinnott-Armstrong W. (1987). Moral realisms and moral dilemmas. J. Philos..

[25] Tasso A., Sarlo M., Lotto L. (2017). Emotions associated with counterfactual comparisons drive decision-making in Footbridge-type moral dilemmas. Motiv. Emot..

[26] Palmiotti G.P., Del Popolo Cristaldi F., Cellini N., Lotto L., Sarlo M. (2020). Framing the outcome of moral dilemmas: Effects of emotional information. Ethics Behav..

[27] McCarthy J., Deady R. (2008). Moral distress reconsidered. Nurs. Ethics..

[28] Dean W., Talbot S.G., Caplan A. (2020). Clarifying the language of clinician distress. JAMA.

[29] Lützen K., Blom T., Ewalds-Kvist B., Winch S. (2010). Moral stress, moralclimate and moral sensitivity among psychiatric professionals. Nurs. Ethics.

[30] Starcke K., Brand M. (2012). Decision making under stress: A selective review. Neurosci. Biobehav. Rev..

[31] Starcke K., Polzer C., Wolf O.T., Brand M. (2011). Does stress alter everyday moral decision-making?. Psych. Neuroendocrinol..

[32] Youssef F.F., Dookeeram K., Basdeo V., Francis E., Doman M., Mamed D. (2012). Stress alters personal moral decision making. Psychneuroendocrinology.

[33] Romero-Rivas C., Rodríguez-Cuadrado S. (2020). Moral decision-makingand mental health during the COVID-19 pandemic. PsyArXiv.

[34] Unützer J., Kimmel R.J., Snowden M. (2020). Psychiatry in the age of COVID-19. World Psychiatry.

[35] Francis K., McNabb C. (2020). Moral Decision-Making during COVID-19: Moral judgments, moralisation, and everyday behavior. Front. Psychol..

[36] Mill J.S. (1863). Utilitarianism.

[37] Singer P. (1972). Famine, Affluence, and Morality.

[38] Scanlon T.M. (2003). The Difficulty of Tolerance: Essays in Political Philosophy.

[39] Brooks S.K., Webster R.K., Smith L.E., Woodland L., Wessely S., Greenberg N., Rubin G.J. (2020). The psychological impact of quarantine and how to reduce it: Rapid review of the evidence. Lancet.

[40] Young L., Camprodon J.A., Hauser M., Pascual-Leone A., Saxe R. (2010). Disruption of the right temporoparietal junction with transcranial magneticstimulation reduces the role of beliefs in moral judgments. Proc. Natl. Acad. Sci. USA.

[41] Schaller U.M., Biscaldi M., Fangmeier T., van Elst L.T., Rauh R. (2019). Intuitive moral reasoning in high-functioning Autism Spectrum disorder: A matter of social schemas?. J. Autism Dev. Disord..

[42] Kurtines W.M., Gewirtz J., Lamb J.L. (1991). Empathy, Social Cognition, and Moral Action. Handbook of Moral Behavior and Development.

[43] Baez S., García A.M., Santamaría-García H., Ibáñez A., Sedeño L., García A. (2017). Moral cognition and moral emotions. Neuroscience and Social Science.

[44] Forbes C.E., Grafman J. (2010). The role of the human prefrontal cortex in social cognition and moral judgment. Annu. Rev. Neurosci..

[45] Del Casale A., Kotzalidis G.D., Rapinesi C., Janiri D., Aragona M., Puzella A. (2017). Neural functional correlates of empathic face processing. Neurosci. Lett..

[46] Eres R., Louis W.R., Molenberghs P. (2018). Common and distinct neural networks involved in fMRI studies investigating morality: An ALEmeta-analysis. Soc. Neurosci..

[47] Greene J.D., Morelli S.A., Lowenberg K., Nystrom L.E., Cohen J.D. (2008). Cognitive load selectively interferes with utilitarian moral judgment. Cognition.

[48] Greene J.D., Nystrom L.E., Engell A.D., Darley J.M., Cohen J.D. (2004). The neural bases of cognitive conflict and control in moral judgment. Neuron.

[49] Greene J.D., Sommerville R.B., Nystrom L.E., Darley J.M., Cohen J.D. (2001). An fMRI investigation of emotional engagement in moral judgment. Science.

[50] Patil I., Zucchelli M.M., Kool W., Campbell S., Fornasier F., Calò M. (2020). Reasoning supports utilitarian resolutions to moral dilemmas acrossdiverse measures. J. Pers. Soc. Psychol..

[51] Moll J., de Oliveira-Souza R., Eslinger P.J., Bramati I.E., Mourão-Miranda J., Andreiuolo P.A. (2002). The neural correlates of moral sensitivity: A functional magnetic resonance imaging investigation of basic and moral emotions. J. Neurosci..

[52] Moran J.M., Young L.L., Saxe R., Lee S.M., O’Young D., Mavros P.L. (2011). Impaired theory of mind for moral judgment in high-functioningautism. Proc. Natl. Acad. Sci. USA.

[53] Bzdok D., Schilbach L., Vogeley K., Schneider K., Laird A.R., Langner R. (2012). Parsing the neural correlates of moral cognition: ALE meta-analysis on morality, theory of mind, and empathy. Brain Struct. Funct..

[54] Hovenkamp-Hermelink J.H., Jeronimus B.F., Spinhoven P., Penninx B.W., Schoevers R.A., Riese H. (2019). Differential associations of locus of control with anxiety, depression and life-events: A five-wave, nine-year study to test stability and change. J. Affect. Disord..

[55] Jordan J., Yoeli E., Rand D. (2020). Don’t get it or don’t spread it? Comparing self-interested versus prosocially framed COVID-19 prevention messaging. PsyArXi..

[56] Pfattheicher S., Nockur L., Böhm R., Sassenrath C., Petersen M.B. (2020). The emotional path to action: Empathy promotes physical distancing during the COVID-19 pandemic. PsyArXiv.

[57] Oosterhoff B., Palmer C. (2020). Psychological correlates of news monitoring, social distancing, disinfecting, and hoarding behaviors among US adolescents during the COVID-19 pandemic. PsyArXiv.

[58] Wang C., Pan R., Wan X., Tan Y., Xu L., Ho C.S., Ho R.C. (2020). Immediate psychological responses and associated factors during the initial stage of the 2019 coronavirus disease (COVID-19) epidemic among the general population in China. Int. J. Environ. Res. Public Health.

[59] Cao W., Fang Z., Hou G., Han M., Xu X., Dong J. (2020). The psychological impact of the COVID-19 epidemic on college students in China. Psychiatry Res..

[60] Elmer T., Mepham K., Stadtfeld C. (2020). Students under lockdown:comparisons of students’ social networks and mental health before and during the COVID-19 Crisis in Switzerland. PLoS ONE.

[61] Gao J., Zheng P., Jia Y., Chen H., Mao Y., Chen S., Dai J. (2020). Mental health problems and social media exposure during COVID-19 outbreak. PLoS ONE.

[62] Ornelas I.J., Tornberg-Belanger S., Balkus J.E., Bravo P., Perez Solorio S.A., Perez G.E., Tran A.N. (2021). Coping With COVID-19: The Impact of the Pandemic on Latina Immigrant Women’s Mental Health and Well-being. Health Educ. Behav..

[63] Li S., Wang Y., Xue J., Zhao N., Zhu T. (2020). The impact of COVID-19epidemic declaration on psychological consequences: A study on active Weibo users. Int. J. Environ. Res. Public Health.

[64] Alyami M., Henning M., Krägeloh C.U., Alyami H. (2021). Psychometric evaluation of the Arabic version of the Fear of COVID-19 Scale. Int. J. Ment. Health Addict..

[65] Sakib N., Bhuiyan A.K.M.I., Hossain S., Al Mamun F., Hosen I., Abdullah A.H. (2020). Psychometric validation of the Bangla Fear of COVID-19 Scale: Confirmatory factor analysis and rsearch analysis. Int. J. Ment. Health Addict..

[66] Satici B., Gocet-Tekin E., Deniz M.E., Satici S.A. (2020). Adaptation of the Fear of COVID-19 Scale: Its association with psychological distress and life satisfaction in Turkey. Int. J. Ment. Health Addict..

[67] Reznik A., Gritsenko V., Konstantinov V., Khamenka N., Isralowitz R. (2020). COVID-19 fear in Eastern Europe: Validation of the Fear of COVID-19 Scale. Int. J. Ment. Health Addict..

[68] Tzur Bitan D., Grossman-Giron A., Bloch Y., Mayer Y., Shiffman N., Mendlovic S. (2020). Fear of COVID-19 Scale: Psychometric characteristics, reliability and validity in the Israeli population. Psychiatry Res..

[69] Huarcaya-Victoria J., Villarreal-Zegarra D., Podestà A., Luna-Cuadros M.A. (2020). Psychometric properties of a Spanish version of the Fear of COVID-19 Scale in general population of Lima, Peru. Int. J. Ment. Health Addict..

[70] Barrios I., Ríos-González C., O’Higgins M., González I., García O., Díaz N.R. (2020). Psychometric properties of the Spanish version of the Fear of COVID-19 Scale (FCV-19S). Int. J. Ment. Health Addict..

[71] Ahorsu D.K., Lin C.Y., Imani V., Saffari M., Griffiths M.D., Pakpour A.H. (2020). The Fear of COVID-19 Scale: Development and initial validation. Int. J. Ment. Health Addict..

[72] Mamun M.A., Griffiths M.D. (2020). First COVID-19 suicide case in Bangladesh due to fear of COVID-19 and xenophobia: Possible suicide prevention strategies. Asian J. Psychiatr..

[73] Duan L., Zhu G. (2020). Psychological interventions for people affected by the COVID-19 epidemic. Lancet Psychiatry.

[74] Huang Y., Zhao N. (2020). Generalised anxiety disorder, depressive symptoms and sleep quality during COVID-19 outbreak in China: A web-based cross-sectional survey. Psychiatry Res..

[75] Dong L., Freedman V.A., de Leon C.F.M. (2020). The association of comorbid depression and anxiety symptoms with disability onset in older adults. Psychosom. Med..

[76] Fiske A., McLennan S., Buyx A. (2021). Ethical insights from the COVID-19 pandemic in Germany: Considerations for building resilient healthcare systems in Europe. Lancet Reg. Health–Eur..

[77] Nuggerud-Galeas S., Oliván Blázquez B., Perez Yus M.C., Valle-Salazar B., Aguilar-Latorre A., Magallón Botaya R. (2020). Factors associated with depressive episode recurrences in primary care: A retrospective, descriptive study. Front. Psychol..

[78] Mak I.W.C., Chu C.M., Pan P.C., Yiu M.G.C., Chan V.L. (2009). Long-term psychiatric morbidities among SARS survivors. Gen. Hosp. Psychiatry.

[79] Lee S.M., Kang W.S., Cho A.R., Kim T., Park J.K. (2018). Psychological impact of the 2015 MERS outbreak on hospital workers and quarantined hemodialysis patients. Compr. Psychiatry.

[80] Morganstein J.C., Ursano R.J. (2020). Ecological disasters and mental health: Causes, consequences, and interventions. Front. Psychol..

[81] Cheung Y.T., Chau P.H., Yip P.S.F. (2008). A revisit on older adults suicides and Severe Acute Respiratory Syndrome (SARS) epidemic in Hong Kong. Int. J. Geriatr. Psychiatry.

[82] Zou P., Sun L., Yang W., Zeng Y., Chen Q., Yang H. (2018). Associations between negative life events and anxiety, depressive, and stress symptoms: A cross-sectional study among Chinese male senior college students. Psychiatry Res..

[83] Adhikari S.P., Meng S., Wu Y.J., Mao Y.P., Ye R.X., Wang Q.Z. (2020). Epidemiology, causes, clinical manifestation and diagnosis, prevention and control of coronavirus disease (COVID-19) during the early outbreak period: A scoping review. Infect. Dis. Poverty.

[84] Orellana C.I., Orellana L.M. (2020). Predictores de síntomas emocionales durante la cuarentena domiciliar por pandemia de COVID-19 en El Salvador. Actual. Psicol..

[85] Ornell F., Schuch J.B., Sordi A.O., Kessler F.H.P. (2020). “Pandemic fear” and COVID-19: Mental health burden and strategies. Braz. J. Psychiatry.

[86] Rodríguez-Rey R., Garrido-Hernansaiz H., Collado S. (2020). Psychological impact and associated factors during the initial stage of the Coronavirus (COVID-19) pandemic among the general population in Spain. Front. Psychol..

[87] Dai Y., Hu G., Xiong H., Qiu H., Yuan X. (2020). Psychological impact of the coronavirus disease 2019 (COVID-19) outbreak on healthcare workers in China. MedRxiv.

[88] Galea S., Merchant R.M., Lurie N. (2020). The mental health consequences of COVID19 and physical distancing: The need for prevention and early intervention. JAMA Med..

[89] Goyal K., Chauhan P., Chhikara K., Gupta P., Singh M.P. (2020). Fear of COVID 2019: First suicidal case in India. Asian J. Psychiatry.

[90] Gaudine A., Thorne L. (2001). Emotion and ethical decision-making in organizations. J. Bus. Ethics.

[91] Ho J.T., Dupasquier J.R., Scarfe M.L., Moscovitch D.A. (2021). Fears of Receiving Compassion from Others Predict Safety Behaviour Use in Social Anxiety Disorder Over and Above Fears of Negative Self-Portrayal. J. Anxiety Disord..

[92] Jizheng H., Mingfeng H., Tengda L., Ake R., Xiaoping Z. (2020). A survey of mental health of medical staff in new-type coronavirus pneumonia hospitals. Chin. J. Ind. Hyg. Occup. Dis..

[93] Xiang Y.T., Yang Y., Li W., Zhang L., Zhang Q., Cheung T., Ng C.H. (2020). Timely mental health care for the 2019 novel coronavirus outbreak is urgently needed. Lancet Psychiatry.

[94] Zhang J., Wu W., Zhao X., Zhang W. (2020). Recommended psychological crisis intervention response to the 2019 novel coronavirus pneumonia outbreak in China: A model of West China Hospital. Precis. Clin. Med..

[95] Kim C.W., Song H.R. (2017). Structural relationships among public’s risk characteristics, trust, risk perception and preventive behavioral intention: The case of MERS in Korea. Crisisnomy.

[96] Tausczik Y., Faasse K., Pennebaker J.W., Petrie K.J. (2012). Public anxiety and information seeking following the H1N1 outbreak: Blogs, newspaper articles, and Wikipedia visits. Health Commun..

[97] Maunder R., Hunter J., Vincent L. (2003). The immediate psychological and occupational impact of the 2003 SARS outbreak in a teaching hospital. CMAJ.

[98] Somma A., Gialdi G., Krueger R.F., Markon K.E., Frau C., Lovallo S., Fossati A. (2020). Dysfunctional personality features, non-scientifically supported causal beliefs, and emotional problems during the first month of the COVID-19 pandemic in Italy. Personal. Individ. Differ..

[99] Bavel J.J.V., Baicker K., Boggio P.S. (2020). Using social and behavioural science to support COVID-19 pandemic response. Nat. Hum. Behav..

[100] Pappa S., Ntella V., Giannakas T., Giannakoulis V.G., Papoutsi E., Katsaounou P. (2020). Prevalence of depression, anxiety, and insomnia among healthcare workers during the COVID-19 pandemic: A systematic review and meta-analysis. Brain Behav. Immun..

[101] Lima C.K.T., Carvalho P.M., Lima I., Nunes J.V.A., Saraiva J.S., Souza R.I., Neto M.L.R. (2020). The emotional impact of coronavirus 2019- Ncov (New Coronavirus Disease). Psychiatry Res..

[102] Román F., Santibáñez P., Vinet E.V. (2016). Uso de las Escalas de Depresión Ansiedad Estrés (DASS-21) como Instrumento de Tamizaje en Jóvenes con Problemas Clínicos. Acta Investig. Psicol..

[103] Sandín B., Valiente R.M., García-Escalera J., Chorot P. (2020). Impacto psicológico de la pandemia de COVID-19: Efectos negativos y positivos en población española asociados al periodo de confinamiento nacional. Rev. Psicopatol. Y Psicol. Clin..

[104] Mertens G., Gerritsen L., Duijndam S., Salemink E., Engelhard I.M. (2020). Fear of the coronavirus (COVID-19): Predictors in an online study conducted in March 2020. J. Anxiety Disord..

[105] Jacobson N.C., Newman M.G. (2017). Anxiety and depression as bidirectional risk factors for one another: A meta-analysis of longitudinal studies. Psychol. Bull..

[106] Jansson-Fröjmark M., Lindblom K. (2008). A bidirectional relationship between anxiety and depression, and insomnia? A prospective study in the general population. J. Psychosom. Res..

[107] Marquez N.M., Littman A.M., Rossi V.E., Everett M.C., Tyagi E., Johnson H.C., Dolovich S.L. (2022). Life Expectancy and COVID-19 in Florida State Prisons. Am. J. Prev. Med..

[108] Fjermestad K.W., Orm S., Silverman W.K., Cogo-Moreira H. (2022). COVID-19-related anxiety is associated with mental health problems among adults with rare disorders. Res. Dev. Disabil..

[109] Bridgland V.M.E., Moeck E.K., Green D.M., Swain T.L., Nayda D.M., Matson L.A., Hutchison N.P., Takarangi M.K.T. (2021). Why the COVID-19 pandemic is a traumatic stressor. PLoS ONE.

[110] Stewart D.E., Appelbaum P.S. (2020). COVID-19 and psychiatrists’ responsibilities: A WPA position paper. World Psychiatry.

[111] McDaid D. (2021). Investing in strategies to support mental health recovery from the COVID-19 pandemic. Eur. Psychiatry.

